# Antifungal Effect of Metabolites from Bacterial Symbionts of Entomopathogenic Nematodes on Fusarium Head Blight of Wheat

**DOI:** 10.3390/jof10020148

**Published:** 2024-02-12

**Authors:** Julius Leumo Kgosiemang, Tshimangadzo Ramakuwela, Sandiswa Figlan, Nicolene Cochrane

**Affiliations:** 1 Agricultural Research Council–Small Grains, Bethlehem 9701, South Africa; 2 Department of Agriculture and Animal Health, University of South Africa, Florida 1710, South Africa; 3 Department of Plant and Soil Sciences, University of Pretoria, Hatfield 0028, South Africa; 4 Agricultural Research Council-Biometry, Pretoria 0001, South Africa

**Keywords:** entomopathogenic nematode, *Fusarium*, *Steinernema*, *Heterorhabditis*, *Xenorhabdus*, *Photorhabdus*

## Abstract

Fungal diseases such as Fusarium head blight (FHB) are significant biotic stressors, negatively affecting wheat production and quality. This study explored the antifungal activity of the metabolites produced by the bacterial symbionts of entomopathogenic nematodes (EPNs) against FHB-causing *Fusarium* sp. *Fusarium graminearum*. To achieve this, the symbiotic bacteria of nine EPN isolates from the EPN collection at the Agricultural Research Council-Small Grains (ARC-SG) were isolated from the cadavers of *Galleria mellonella* (Lepidoptera: *Pyralidae*) larvae after infection with EPNs. Broth cultures (crude) and their supernatants (filtered and autoclaved) of each bacterial isolate were used as bacterial metabolite treatments to test their inhibitory effect on the mycelial growth and spore germination of *F. graminearum*. Mycelial growth inhibition rates varied among both bacterial isolates and treatments. Crude metabolite treatments proved to be more effective than filtered and autoclaved metabolite treatments, with an overall inhibition rate of 75.25% compared to 23.93% and 13.32%, respectively. From the crude metabolite treatments, the *Xenorhabdus khoisanae* SGI 197 bacterial isolate from *Steinernema beitlechemi* SGI 197 had the highest mean inhibition rate of 96.25%, followed by *Photorhabdus luminescens* SGI 170 bacteria isolated from *Heterorhabditis bacteriophora* SGI 170 with a 95.79% mean inhibition rate. The filtered metabolite treatments of all bacterial isolates were tested for their inhibitory activity against *Fusarium graminearum* spore germination. Mean spore germination inhibition rates from *Xenorhabdus* spp. bacterial isolates were higher (83.91 to 96.29%) than those from *Photorhabdus* spp. (6.05 to 14.74%). The results obtained from this study suggest that EPN symbiotic bacterial metabolites have potential use as biological control agents of FHB. Although field efficacy against FHB was not studied, the significant inhibition of mycelial growth and spore germination suggest that the application of these metabolites at the flowering stage may provide protection to plants against infection with or spread of *F. graminearum*. These metabolites have the potential to be employed as part of integrated pest management (IPM) to inhibit/delay conidia germination until the anthesis (flowering stage) of wheat seedlings has passed.

## 1. Introduction

Wheat is a key agricultural commodity globally with widespread consumption and usage [1,2]. An estimated 20% of the total calories that humans consume come from wheat, and it gives more protein than other food sources [3]. To cater to the needs of the growing human population, there is a demand for wheat research and breeding to speed up the genetic gain and improve wheat yield and quality [3,4]. The reduced availability of good farmland and the effect of climate change, among other irregular abiotic and biotic stresses, continue to threaten wheat production globally [5]. Of all the important diseases of wheat, fungal diseases pose a significant threat for widening the gap between actual and attainable yield [6]. Figueroa et al. [5] reviewed some of the causal fungal wheat diseases, of which wheat rusts, blotch diseases, and Fusarium head blight (FHB) are significant.

Fusarium head blight, a significant floral disease of cereals and poses serious health hazard to humans and animals through the contamination of grain with harmful mycotoxins [7,8,9]. Fusarium head blight occurs in most wheat cultivars, and infections are mainly influenced by air, temperature, and atmospheric humidity during flowering and the early stages of seedling development [10]. This disease is caused mainly by the Ascomycete fungus *Fusarium graminearum* [5]. Fusarium head blight disease leads to the early aging of the wheat heads, resulting in the reduction in both yield and grain quality by up to 80% [11]. Moreover, the production of mycotoxins such as zearalenone, zearalene, deoxynivalenol (DON) or nivalenol, T-2 toxin, and diacetoxyscirpenol can cause serious harm to both humans and animals [7,12]. *Fusarium graminearum* along with *F. fujikuroi* species complexes are the major toxin producers in the genus *Fusarium* [13].

In order to control FHB, chemical pesticides have been used for years [14]. Different active molecules, such as triazoles and imidazoles, have been reported for their efficacy against FHB-causing *Fusarium* spp. [15]. Additionally, wheat farmers have been applying benzimidazole chemical pesticides, mainly carbendazim, to control FHB in the recent decades [16] due to their low price compared to other classes of chemical pesticides [17]. Although chemical pesticide application forms a critical part of integrated FHB management [18], the foliar application of fungicides at anthesis (the optimum growth stage for chemical pesticides application for FHB control [19]) might provide some protection, but these pesticides destroy the natural antagonists of plant pathogens and induce pathogen populations’ resistance to chemicals [20]. Furthermore, resistance to carbendazim can enhance DON biosynthesis in infected wheat [16]. On the other hand, the quinone outside inhibitor (QoI) class of chemical pesticides, including the strobilurins, can result in increased DON accumulation, even though they partially control FHB incidence [17]. The use of chemicals and genetically resistant cultivars is continuously challenged by the development of pathogen resistance/virulence, while harsh pesticides pose environmental and human/animal health risks. There are currently no effective FHB control strategies available for farmers [21].

Since the concept of integrated pest management (IPM) is principled on minimizing the dependence on chemical pesticides [22], which are associated with many health and environmental issues, O’Brien et al. [23] mentioned that the level of disease suppression achieved via the application of biocontrol agents to a plant can be the same as that achieved via application of a chemical pesticide. Entomopathogenic nematodes (EPNs) are obligate parasites of insects [24]. Out of all the EPN families, Steinernematidae and Heterorhabditidae have gained more attention due to their many attributes as effective biological control agents [25]. Entomopathogenic nematodes from the genera *Steinernema* and *Heterorhabditis* have been commercialized in several continents [26] and used to control a wide range of agriculturally important insect pests, aided by their bacterial symbionts [27,28,29]. In contrast to their obvious potential, the commercial successes of EPNs’ application are limited [30] by a number of biotic and abiotic factors that affect EPN’s pest control efficacy [31], especially for foliar application.

The genera *Xenorhabdus* and *Photorhabdus* from the *Morganellaceae* family are entomopathogenic bacteria that cause septicemia and toxemia in their insect hosts [32]. In nature, *Photorhabdus* and *Xenorhabdus* spp. live in symbiosis with EPNs of the family Heterorhabbditidae and Steinernematidae, respectively [33]. These bacterial symbionts are released from their respective EPN’s infective juvenile into the insect’s hemocoel, where they reproduce and kill the insect host rapidly within 48 h [34]. *Photorhabdus* and *Xenorhabdus* spp. are potent producers of structurally diverse compounds that are important during their mutualistic lifestyle in symbiosis with nematodes, the infection of the insect host, and the protection of the host cadaver against competitors [35]. They produce a great number of secondary metabolites, including lipases, phospholipases, proteases, and peptides, which are assumed to produce novel natural compounds with diverse biological activities [36]. The secondary metabolites of *Photorhabdus* and *Xenorhabdus* bacteria have demonstrated inhibitory effects against various fungal plant pathogens [37,38,39] and are said to have no phytotoxic effects when applied to various plant species in greenhouses [39]. This current study is one of the first to test the efficacy of EPNs’ symbiotic bacterial metabolites against FHB-causing *Fusarium* sp. *F. graminearum* group II (also known as *Gibberellazeae* sexual stage), which is both the main and most virulent *Fusarium* species causing FHB [40] and the major toxin (DON) producer [41]. This study evaluated antifungal activity of metabolites produced by the bacterial symbionts of EPNs against *F. graminearum* mycelial growth and spore germination.

## 2. Materials and Methods

### 2.1. Fusarium sp. Cultures

*Fusarium graminearum* cultures (isolate D52) were provided by the Fusarium laboratory at ARC-SG, and they were successively subcultured onto plates of potato dextrose agar (PDA) (Merck Co., Darmstadt, Germany) and mung bean agar [MBA-mung bean extracts + bacteriological agar (LAB M Limited, Bury, UK)]. The plates were then incubated at 25 ± 1 °C for four days and seven days, respectively. After incubation, Fusarium spores were confirmed under a light microscope (Model U-LHLEDC, Olympus Co., Tokyo, Japan) at 40× magnification, and plates were kept at ±10 °C until later use.

### 2.2. Isolation of EPNs Symbiotic Bacteria

Nine indigenous EPN isolates (Table 1) from the EPN collection at ARC-SG were used for this study. Isolation of the symbiotic bacteria was achieved by using a combination of the protocols of Kaya and Stock [42] as well as Muangpat et al. [43], with some modifications. Briefly, for each isolate, three last-instar *Galleria mellonella* (Linnaeus) (Lepidoptera: Pyralidae) larvae were infected and incubated for 48 h at 25 ± 1 °C in the dark. Under a laminar flow hood, symbiotic bacteria were isolated from the hemolymph by dissecting the cadavers of *G. mellonella* larvae with a sterile scalpel blade. The hemolymph inoculum were then streaked onto selective medium. MacConkey agar (Merck Co., Darmstadt, Germany) medium was used for *Photorhabdus* spp. isolated from *Heterorhabditis* EPN isolates, and nutrient bromothymol blue triphenyltetrazolium chloride agar (NBTA-nutrient agar, 0.0025% bromothymol blue and 0.004% triphenyltetrazolium chloride) medium was used for *Xenorhabdus* spp. isolated from *Steinernema* isolates [44]. Inoculated plates were incubated at 25 ± 1 °C for 72 h in the dark. Morphological identification of bacterial colonies was achieved by visually observing characteristics of the colonies and by making a slide and observing cell morphology as described by Akhurst [45]. To obtain pure bacterial cultures, single colonies of the desired morphology and characteristics were picked and plated successively onto new MacConkey agar or NBTA plates to produce a pure culture. For stock preservation, bacteria were suspended in 15% glycerol and stored at −80 °C until needed for experiments [36].

### 2.3. Production of Bacterial Metabolites

Production of bacterial metabolites was performed as described by Eroglu et al. [36]. Each bacterial isolate was grown on MacConkey agar/NBTA for 72 h at 25 ± 1 °C in the dark. Then, for each isolate, a loop of bacterial cells from a single colony was harvested and transferred into 100 mL Tryptic Soy Broth (TSB) (Merck KGaA, Darmstadt, Germany) in a 250 mL Erlenmeyer flask. Three flasks were inoculated as replicates for each isolate. The liquid cultures were covered in foil and incubated on a rotary shaking incubator at 180 rpm for seven days at 25 ± 1 °C.

### 2.4. Preparation of Metabolite Treatments

Seven-day-incubated bacterial liquid cultures were used as the first treatment, and they are referred to as crude metabolite treatments. The second type of bacterial metabolites treatment was prepared via centrifuging (Hermle Z200A, Hermle, Germany) bacterial liquid cultures at 6000 rpm for 20 min, followed by filtering the supernatants through 0.22 μm Millipore filter discs (Merck KGaA, Darmstadt, Germany), and they are referred to as filtered metabolite treatments. The last type of treatment was prepared by autoclaving the supernatants at 121 °C for 15 min, and they are referred to as autoclaved metabolite treatments. All three types of bacterial metabolite treatments were transferred into 50 mL falcon tubes and kept at 10 ± 1 °C until later use (overnight for mycelial growth experiments and 4 days for spore germination experiments).

### 2.5. Efficacy of Bacterial Metabolite Treatments on F. graminearum Mycelial Growth

All three types of bacterial metabolite treatments were used to test their efficacy on *F. graminearum* mycelial growth. Bacterial metabolite treatments were incorporated into PDA at 20% following the procedure set out by Hazir et al. [39]. Before autoclaving PDA, 20% of the prescribed distilled water was omitted when preparing the media for subsequent addition of metabolite treatment suspensions. After preparation, 40 mL of the media was poured into 100 mL Erlenmeyer flasks and autoclaved. After autoclaving the media, the flasks were allowed to cool to 45–50 °C by placing them in a water bath, then 10 mL (20%) of bacterial metabolite treatments (crude, filtered and autoclaved) from each of the nine isolates was added and mixed thoroughly before pouring into three 65 mm Petri dish plates. Sterile distilled water was added to control treatments. Using a sterile cork-borer, the center of each PDA plate was welled (5 mm in diameter). These wells were plugged with 5 mm diameter pieces of *F. graminearum*-infected PDA (previously incubated for 4 days at 25 ± 1 °C). After filling the wells, the plates were sealed and incubated at 25 ± 1 °C for 7 days in the dark. Two growth diameters (measurements taken perpendicular to each other using a ruler) were measured per plate after 3- and 7-day incubation periods. The inhibition rate of mycelial growth was calculated using the formula: inhibition rate = 100 × (colony diameter in control−colony diameter in treatment)/colony diameter in control [39]. The experiment had three replicates per treatment, and it was conducted three times on different dates with a different batch of bacterial metabolites.

### 2.6. Efficacy of Bacterial Metabolites against F. graminearum Spore Germination

Suppression of spore germination was performed as described by Hazir et al. [39]. Fusarium cultures were subcultured onto MBA plates (90 mm) and incubated at 25 ± 1 °C for 7 days, after which spores were harvested from the plates by suspending them in sterile water. Then, 0.1 mL of the conidia suspensions was added to 0.7 mL potato dextrose broth (PDB) (Sigma-Aldrich, Saint Louis, MI, USA) in 2 mL Eppendorf tubes containing 0.2 mL (20% *v*/*v*) bacterial metabolite treatments of each isolate. For the control, PDB (0.9 mL) alone and 0.1 mL conidial suspensions without bacterial metabolites were used. The tubes were incubated at 25 ± 1 °C for 3 days followed by loading spore suspensions on the hemocytometer slide and counting the first 100 conidia under the compound microscope (Model U-LHLEDC, Olympus Co., Tokyo, Japan) to determine percentage spore germination. The inhibition rate of spore germination was calculated by using the formula: inhibition rate = 100 × (spores germinated in control−spores germinated in treatment)/spores germinated in control [39]. Each treatment had three replicates, and the experiment was conducted three times at different dates with a different batch of bacterial metabolites.

### 2.7. Statistical Analysis

Analysis of variance (ANOVA) (α = 0.05) was used to detect the significance of the effects of the treatments on mycelial growth and spore germination. Trial repeats of both experiments were assessed to determine whether there were significant differences between experiment repeats. For the spore germination experiment, the data were combined, as there were no significant differences between trials (*p* > 0.1215). The standardized residuals were normally distributed (Shapiro–Wilk test); therefore, the means of spore germination percentages were separated using Fisher’s unprotected *t*-test (least significant difference (LSD)) at α = 0.05 [49]. For mycelial growth, growth inhibition data from repeated trials were pooled. The trials were checked for homogeneity using Levene’s test and Bartlett’s test before pooling the data. The means of the growth inhibition diameters were separated using Fisher’s unprotected *t*-test (least significant difference (LSD)) at α = 0.05 [49].

## 3. Results

### 3.1. Efficacy of Bacterial Metabolites on F. graminearum Mycelial Growth

**Figure 1:**
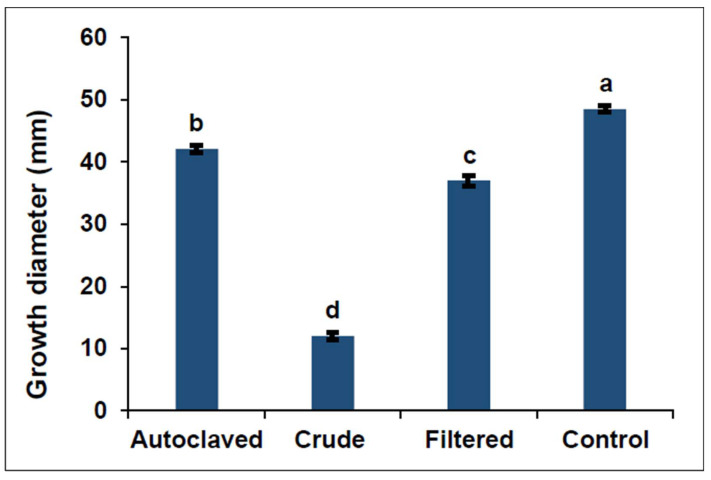
Mean mycelia growth diameter (mm) on potato dextrose agar treated with different bacterial metabolite treatments (autoclaved, crude and filtered) produced by different isolates compared to control. Different letters above standard error bars indicate significant differences between treatments (α = 0.05).

**Figure 2:**
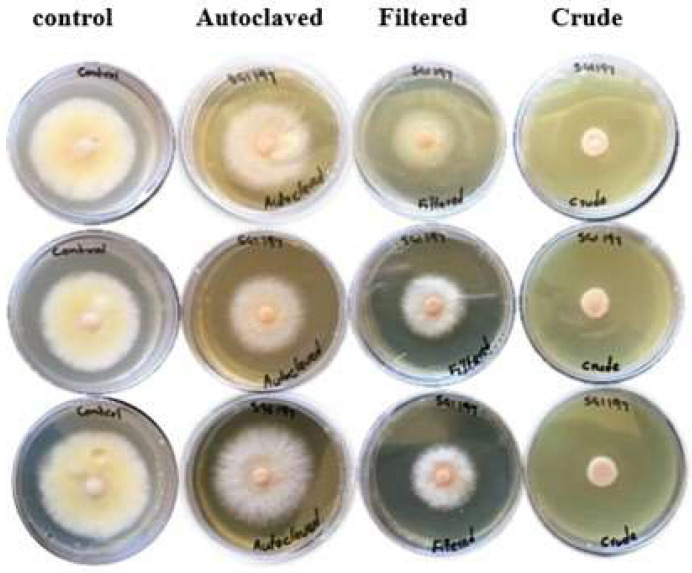
*Fusarium graminearum* mycelia growth, showing growth inhibition of the different treatments compared to control, three days post inoculation.

The ANOVA presented varying significant inhibitions of mycelial growth from the three different metabolite treatments (crude, filtered, and autoclaved metabolites) of each isolate. Crude metabolite treatments exhibited the highest growth inhibition activity when compared to the other two metabolite treatments (filtered metabolites and autoclaved metabolites), while autoclaved metabolites exhibited the lowest growth inhibition activity when compared to the other two metabolite treatments (crude metabolites and filtered metabolites) in all bacterial isolates (F = 2855.75; df = 3; *p* < 0.0001) (Figure 1 and Figure 2). The mean crude metabolite inhibition rates of all isolates ranged from 64.12 to 86.76%, while the mean filtered metabolite inhibition rates ranged from 17.65 to 60.88% and autoclaved metabolites mean inhibition rates ranged from 0 to 21.72% after three days of incubation. After seven days of incubation, mean inhibition rates ranged from 42.88 to 96.25% for crude metabolites, 0 to 55.9% for filtered metabolites, and 0 to 5.4% for autoclaved metabolites (Table 2). From the crude metabolite treatments, *X. khoisanae* SGI 197 crude metabolites had the highest inhibition rate of 96.25%, followed by *P. luminescens* SGI 170 crude metabolites with a 95.79% inhibition rate.

### 3.2. Efficacy of Bacterial Metabolites on F. graminearum Spore Germination

**Figure 3:**
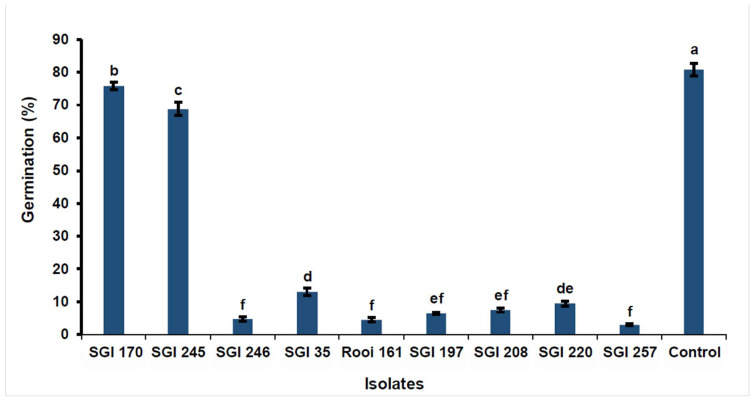
Mean spore germination percentages in potato dextrose broth treated with 20% bacterial metabolites of different entomopathogenic nematode symbiotic bacterial metabolites compared to control. Different letters above standard error bars indicate significant differences between treatments (α = 0.05).

For spore germination experiments, three trials were conducted on different dates, and all three trials exhibited a similar pattern of the inhibition of spore germination after the ANOVA of the results (F = 2.15; df = 2; *p* = 0.1258). The control exhibited the highest percentage (80.78%) of spore germination, which was the highest of all the isolates tested (Figure 3). *Xenorhabdus* isolates had higher inhibition than *Photorhabdus* isolates (F = 407.46; df = 9; *p* < 0.0001). Amongst the *Xenorhabdus* isolates, *Xenorhabdus* sp. SGI 257 had the highest spore germination inhibition percentage (F = 540.30; df = 7; *p* < 0.0001). Table 3 illustrates the inhibition rate (%) of the bacterial metabolite treatments when compared to the control.

## 4. Discussion

The results show different levels of inhibition of the mycelial growth and spore germination of *F. graminearum* among all tested isolates and treatments. The level of inhibition varied significantly among isolates and treatments. Hazir et al. [39] reported similar results when they investigated the relative potency of secondary metabolites in various *Photorhabdus* and *Xenorhabdus* supernatants for diverse fungal phytopathogens. Additionally, metabolites from EPNs symbionts *Photorhabdus* and *Xenorhabdus* have also been reported to have varying antifungal activities [38,39,50]. However, this current study is one of the first to test and show the effectiveness of EPN symbiotic bacterial metabolites against FHB-causing *Fusarium* sp. *F. graminearum* group II.

Shan et al. [51] mentioned that the antimicrobial activity of symbiotic bacteria derived from EPNs against the mycelial growth of plant pathogens depends on the species of symbiotic bacteria. *Photorhabdus luminescens* SGI 170 followed *X. khoisanae* SGI 197 as the second highest performing isolate, with a 95.79% mean inhibition rate after 7 days’ incubation. In addition to this South African-based study, *Xenorhabdus* spp. displayed antifungal activity against a number of fungal phytopathogens other than *F. graminearum* in other parts of the world [52,53,54,55]. Indian *Xenorhabdus* species (*Xenorhabdus assam*, *X. indica*, and *X. Gujarat*) displayed antifungal activity against *Fusarium oxysporum*, *Macrophomina phaseolina*, *Sclerotium rolfsii*, and *Rizoctonia solani* [38]. From their results, *X. assam* achieved a 78.1–82.2% inhibition rate of *S. rolfsii*, *F. oxysporum* and *R. solani* and complete inhibition of *M. phaseolina*, while *X. indica* had the strongest activity against *F. oxysporum* and the weakest activity against *M. phaseolina*. Chinese *Xenorhabdus* sp. *X. nematophila* displayed antifungal activity against maize fungal pathogens *Bipolarismaydis* and *Curvularialunata* with inhibition rates of 66.7% and 69.1%, respectively [34]. The results we obtained from our *Xenorhabdus* sp. SGI 197 crude metabolite treatments seem to support antifungal activity results obtained in other parts of the world but with a higher inhibition of 96.25% against *F. graminearum*.

*Photorhabdus* spp. isolated from *Heterorhabditis* spp. are known as key producers of trans-cinnamic acid (TCA) [50]. Trans-cinnamic acid is said to have antifungal properties [39,50,51]. Lalramchuani et al. [56] reported raising the inhibition rate using a *Photorhabdus* sp. *Photorhabdus luminescens* subsp. Akhurst [45], which, on the other hand, displayed a 50 to 60% inhibition rate of *Fusarium oxysporum* mycelial growth after 48 h, which went up to a 76–79% inhibition rate after 96 h, and raised again to 87% after 192 h of incubation. Similarly, in this study, crude metabolite treatment inhibition rates of both *Photorhabdus* isolates *P. luminescens* SGI 170 and *Photorhabdus* sp. SGI 245 increased from 73.82% to 95.79% and 86.76% to 88.39% after 3 days and 7 days of incubation, respectively. The increase in the inhibition rate of these two isolates may be related to the antifungal compound TCA. An increase in inhibition rate was also achieved with two *Xenorhabdus* isolates [*Xenorhabdus* sp. SGI 197 (83.53 to 96.25%) and *Xenorhabdus* sp. SGI 257 (74.12 to 85.77%)], while other *Xenorhabdus* isolates had displayed a decrease in inhibition rate after 3 days and 7 days of incubation. The varied antagonistic effects between *Xenorhabdus* isolates may be attributed to the differences in the production levels of their antifungal compounds.

Overall, crude treatments were the most effective against *F. graminearum* mycelial growth. The overall mean inhibition rate of crude treatments was 75.25%, which was higher than that of filtered treatments (23.93%) and autoclaved treatments (13.32%). The lower inhibition activity of autoclaved treatments could be attributed to the denaturation of the enzymes responsible for the synthesis of secondary metabolites. The enzymes involved in production of these metabolites are polyketide synthetases (PRSs), nonribosomal peptide synthetases (NRPSs), and other similar enzymes [50]. Wang et al. [34] also reported a decline in the inhibition activity of the metabolites against *Bipolaris maydis* after their exposure to high temperatures (50 °C and 100 °C). However, the same metabolites did not lose their inhibition activity against *Curvularia lunata* after their exposure to high temperatures. Additionally, Hazir et al. [39] demonstrated that the metabolites produced by *Xenorhabdus* sp. *X. szentirmaii* did not lose their antifungal activity after autoclaving them at 121 °C for 15 min. From their results, autoclaved metabolite treatment from *X. szentirmaii* displayed similar inhibition activity against *Monilinia fructicola* as the filtered metabolites treatment but higher inhibition activity against *Glomerella cingulata* than the filtered metabolite treatment. Nevertheless, it is worth noting that these studies, compared to the current study, did not compare autoclaved metabolites to crude metabolites. This is contrary to our results where we found that autoclaved metabolites lost inhibitory activity against *F. graminearum*. Metabolites from *Xenorhabdus* and *Photorhabdus* differ in stability when exposed to high temperatures, and their antifungal activity seems to depend on the species of fungus being treated.

Isolates from the genus *Xenorhabdus* displayed higher inhibition rates against *F. graminearum* spore germination than isolates from the genus *Photorhabdus*. Over 83% inhibition of *Fusarium* spore germination was achieved with all isolates from the genus *Xenrhabdus*, while that of *Photorhabdus* was below 15%. Among the *Xenorhabdus* isolates, *Xenorhabdus* sp. SGI 257 had the highest inhibition rate (96.29%) of spore germination. Hazir et al. [39] reported a similar trend when they tested seven bacterial isolates (four *Xenorhabdus* spp. and three *Photorhabdus* isolates) to determine the inhibitory effect of metabolites on the spore germination of *Fusicladium carpophilum* and *Fusicladium effusum*. From their results, germination was lower in treatments from *Xenorhabdus* spp. than from *Photorhabdus* spp. for both *F. carpophilum* and *F. effusum*, except for metabolites from *Xenorhabdus nematophila*, which displayed intermediate results when tested on *F. carpophilum* spores. In contrast, the inhibitory effect of *Xenorhabdus* and *Photorhabdus* metabolites seemed to depend on the isolate tested, not on the genus when tested against *Pythium myriotylum* spores [51]. Spore germination is a critical step in the development of FHB disease in wheat seedlings. From our results, bacterial metabolites from the genus *Xenorhabdus* have the potential as candidates for use in integrated FHB management.

## 5. Conclusions

We found that the bacterial metabolites produced by the bacterial symbionts of EPNs had an antifungal effect on two important development stages (mycelial growth and spore germination) of FHB-causing *Fusarium* sp. *F. graminearum*. Overall, crude treatments were the most effective at restricting *F. graminearum* mycelial growth, and isolates from the genus *Xenorhabdus* displayed higher inhibition rates of *F. graminearum* spore germination than isolates from the genus *Photorhabdus*. Although field efficacy against FHB was not studied, the significant inhibition of mycelial growth and spore germination suggests that the application of bacterial metabolites at the flowering stage may provide protection for plants against infection with or spread of *F. graminearum* by preventing spore germination and mycelia growth. Further research is ongoing to isolate, identify, and characterize the metabolites produced by the EPNs’ symbiotic bacteria and to prove that their application will be safe for nontarget organisms, plants, and the environment before they are used as bio-fungicides. Moreover, studies are needed to evaluate the efficacy of these metabolites under field conditions to address their effects on DON accumulation and beneficial organisms. A locally produced bio-fungicide of this nature may be more economical in price, especially using the crude metabolite type, which is the most effective and requires less processing. This will have far-reaching impacts, especially for resource-poor farmers.

## Figures and Tables

**Table 1:** Selected indigenous entomopathogenic nematode isolates for isolation of their symbiotic bacteria.

Isolate	Nematode	Symbiotic Bacteria
SGI 35	*Steinernema innovationi*	*Xenorhabdus* sp.
SGI 170	*Heterorhabditis bacteriophora*	*Photorhabdus luminescens* [46,47]
SGI 197	*Steinernema beitlechemi*	*Xenorhabdus khoisanae* [48]
SGI 208	*Steinernema* sp.	*Xenorhabdus* sp.
SGI 220	*Steinernema* sp.	*Xenorhabdus* sp.
SGI 245	*Heterorhabditis bacteriophora*	*Photorhabdus* sp.
SGI 246	*Steinernema biddulphi*	*Xenorhabdus* sp.
SGI 257	*Steinernema* spp.	*Xenorhabdus* sp.
ROOI 161	*Steinernema khoisanae*	*Xenorhabdus* sp.

**Table 2:** Mycelial growth inhibition rates (%) of all isolates in both incubation periods.

	3 Days’ Incubation			7 Days’ Incubation		
Isolate	Crude	Filtered	Autoclaved	Crude	Filtered	Autoclaved
*Xenorhabdus* sp. SGI 35	77.65	58.82	14.30	47.33	29.59	4.52
*Photorhabdus luminescens* SGI 170	73.82	60.88	7.35	95.79	55.90	1.69
*Xenorhabdus khoisanae* SGI 197	83.53	42.06	21.72	96.25	15.49	5.37
*Xenorhabdus* sp. SGI 208	76.18	38.53	18.27	61.66	2.06	1.69
*Xenorhabdus* sp. GI 220	68.82	39.12	11.76	42.88	2.43	4.52
*Photorhabdus* sp. SGI 245	86.76	17.65	0.00	88.39	28.37	1.69
*Xenorhabdus* sp. SGI 246	64.12	20.29	0.00	54.30	0.00	0.00
*Xenorhabdus* sp. SGI 257	74.12	30.00	15.88	85.77	14.42	0.85
*Xenorhabdus* sp. ROOI 161	81.76	31.18	9.71	81.46	1.87	0.00

**Table 3:** Mean inhibition rate (%) of *Fusarium graminearum* spore germination. Inhibition rate = 100 × (spores germinated in control–spores germinated in treatment)/spores germinated in control [39].

Isolate	Spore Germination Percentage	Inhibition Rate
*Xenorhabdus* sp. SGI 35	13.00	83.91
*Photorhabdus luminescens* SGI 170	75.89	6.05
*Xenorhabdus khoisanae* SGI 197	6.44	92.02
*Xenorhabdus* sp. SGI 208	7.44	90.78
*Xenorhabdus* sp. SGI 220	9.50	88.23
*Photorhabdus* sp. SGI 245	68.86	14.74
*Xenorhabdus* sp. SGI 246	4.78	94.09
*Xenorhabdus* sp. SGI 257	3.00	96.29
*Xenorhabdus* sp. ROOI 161	4.56	94.36

## Data Availability

Data are contained within the article.
